# Deciphering global patterns of marine microbial community assembly and network stability

**DOI:** 10.1128/msystems.00470-26

**Published:** 2026-06-22

**Authors:** Pranathi Ravikumar, Aarti Ravindran, Karthik Raman

**Affiliations:** 1 Department of Biotechnology, Bhupat and Jyoti Mehta School of Biosciences, Indian Institute of Technology Madras 37268, Chennai, Tamil Nadu, India; 2 Centre for Integrative Biology and Systems mEdicine (IBSE), Wadhwani School of Data Science and AI, IIT Madras 37268, Chennai, Tamil Nadu, India; 3 Department of Data Science and AI, Wadhwani School of Data Science and AI, Indian Institute of Technology Madras 37268, Chennai, Tamil Nadu, India; University of California, Davis, Davis, California, USA

**Keywords:** marine microbiome, community assembly, co-occurrence networks, microbial diversity, network stability

## Abstract

Microbial community ecology seeks to unravel the patterns and processes that govern the diversity, assembly, and functional stability of microbial assemblages across global ecosystems. In recent years, the increased availability of sequencing data from large-scale ocean microbiome projects has made it feasible to study microbial community assembly and its underlying mechanisms across global marine environments. In this study, we have investigated species richness patterns, community assembly mechanisms, and interaction patterns of marine bacterial communities by analyzing 16S ribosomal RNA amplicon sequencing data from 4,611 samples collected from ocean microbiome projects. Using neutral community models, the iCAMP framework, and co-occurrence network analyses, we showed that stochastic processes drive microbial community assembly across all latitude zones. In the polar zone, dispersal limitation was the primary driver of community assembly, compared with temperate and tropical communities, where dispersal limitation and selection played an important role. Polar microbial communities exhibited the highest modularity and network robustness, but were more vulnerable to hub removal. Although previous studies have attributed the higher stability of polar communities to environmental filtering, our analyses reveal that the resilience of the community is dependent on a few central taxa. By classifying the genera as generalists and specialists, we further highlight the role played by the specialist taxa in maintaining the stability of the marine microbial community, especially under the pressures of climate change and global warming. In general, our findings offer a latitudinal perspective on the stability of the ocean bacterial community, with implications for understanding their responses to environmental disturbances.

**IMPORTANCE:**

Marine microbes play a vital role in sustaining food webs, cycling nutrients, and regulating Earth's climate. However, we still lack a global understanding of how these microbial communities form, interact, and remain stable under environmental change. By analyzing over 4,600 ocean samples from across the globe, using integrative approaches like neutral models, iCAMP, and network analysis, we have dissected the processes driving bacterial community assembly across different latitude zones and identified that the relative contributions of deterministic and stochastic processes vary significantly. This latitudinal variation in the assembly mechanisms highlights the complexity of the dynamics of the bacterial community in the ocean. Importantly, identifying the pivotal role of specialist taxa in upholding community stability underlines the vulnerability of these ecosystems to disturbances that could disrupt key microbial interactions. Understanding these microbial dynamics is critical for conserving ocean health and sustaining the processes that govern global ecosystems.

## INTRODUCTION

Microbial community assembly is governed by ecological, evolutionary, and environmental factors that shape community structure and diversity. Large-scale ocean microbiome projects, including the Tara Oceans Project (1), Malaspina Expedition (2, 3), Global Ocean Sampling (4, 5), and Bio-GO-SHIP (6), have revolutionized our understanding of microbial life in the oceans. These initiatives have enabled public access to microbial community data across spatial and environmental gradients, supporting global-scale analysis of the ocean microbiome.

To investigate the ecological mechanisms underlying community assembly, researchers employ conceptual frameworks and statistical methods that differentiate between deterministic (niche-driven) and stochastic (neutral) processes (7–9). Deterministic processes, such as environmental selection and species interactions, structure communities by selecting traits suited to particular environmental conditions. On the other hand, stochastic processes include ecological drift, dispersal limitation, and historical contingencies, resulting in community patterns that arise from random events and colonization. Models, such as the neutral community model (10) and the iCAMP (infer community assembly mechanisms by phylogenetic bin-based null model analysis) framework (11), enable quantitative estimation of the contribution of these processes in driving community assembly.

While community assembly models uncover abiotic drivers of community composition, they cannot capture the interactions of taxa within the communities. Hence, co-occurrence network analysis can be a powerful tool in microbial ecology. Co-occurrence networks are constructed by identifying statistically significant correlations between taxa across samples, enabling the inference of potential ecological interactions (12). The structure of these networks can be quantified using network properties such as modularity, degree distribution, clustering coefficient, and natural connectivity. These properties can reveal emergent properties such as stability, robustness, and keystone taxa in the communities (13).

These network topologies often explain the ecological roles played by the taxa. For example, some genera may act as generalists and have high prevalence across environments. On the other hand, they may be specialists, having niche-specific prevalence. These patterns can be used to understand how microbial ecosystems respond to perturbations such as warming, acidification, and nutrient fluctuations (14, 15).

One of the most widely used methods to characterize marine microbial communities in such studies is 16S ribosomal RNA (rRNA) gene amplicon sequencing (16–18). This technique targets conserved regions of the rRNA gene to profile taxonomic composition at relatively low costs and high throughput (19). It can be used to estimate microbial diversity and biogeographic patterns to understand ecological processes in these communities (20). However, it is important to note that 16S rRNA gene sequencing primarily captures the prokaryotic community (bacteria and archaea) and does not represent the full microbial community, which also includes eukaryotic microorganisms (21).

We have utilized 16S rRNA amplicon sequencing data from the Australian Microbiome Project (22), Earth Microbiome Project (23, 24), Tara Oceans Project (1), Global Ocean Sampling (4), Bio-GO-SHIP (6), and Malaspina Expedition (2, 3, 25). We have investigated the ecological mechanisms governing community assembly processes in the ocean microbial community using the Sloan neutral community model (NCM) and the iCAMP framework. Furthermore, co-occurrence networks were constructed and have been used to uncover structural features and keystone taxa. The microbes in these networks were classified as generalists and specialists based on their prevalence to evaluate their topological roles and contributions to network robustness.

Through this integrative framework, the study aims to provide a comprehensive understanding of the ecological processes structuring global marine microbial communities, focusing on how latitude-mediated environmental gradients influence the presence of taxa within these communities, and how they interact and function as a collective ecological network.

## MATERIALS AND METHODS

### Data retrieval and preprocessing

A total of 4,611 16S amplicon sequencing samples were obtained from multiple ocean microbiome projects, including Tara Oceans Project (1), Malaspina Expedition (2,3), Global Ocean Sampling (4), and Bio-GO-SHIP (6). Additionally, marine samples from the Australian Microbiome Project (22) and the Earth Microbiome Project were incorporated (23, 24) (Table 1). The number of samples taken from each of these projects has been mentioned in (see Table S1 at https://doi.org/10.5281/zenodo.20175118). The sample locations have been inferred from the latitude and longitude data and incorporated into the metadata file (see File S1, Fig. S1, and Table S1 at https://doi.org/10.5281/zenodo.20175118).

**TABLE 1:** Number of samples across different microbiome projects

Project	Samples
Australian Microbiome	2,368
Bio-GO-SHIP	454
Earth Microbiome Project	1,306
Global Ocean Sampling	47
Malaspina Expedition	274
Tara Oceans Project	134

All the data sets in our study had information on the latitude and longitude coordinates, the ocean basin (assigned based on latitude coordinates), and the sample origin; these covariates were included in our analyses. Latitude was further used to classify samples into three broad climatic zones: tropical (23.5°N to 23.5°S), temperate (23.5°–66.5° in both hemispheres), and polar (>66.5°N and <66.5°S). To minimize misclassification of samples located near zone boundaries and reduce transitional environmental effects, samples within a 5° buffer of each boundary were excluded from zone-based analyses. This ensured clearer separation of ecological regimes and improved robustness of downstream comparisons. The sample origin was categorized into four groups based on environmental context: marine (open water/seawater samples), coral, sponge, and marine sediment samples. These classifications were based on the sample descriptions and associated metadata provided in the original data sets. The samples have been collected between 2002-2023 (as mentioned in Files S1 and S3 at https://doi.org/10.5281/zenodo.20175118).

The quality filtering and preprocessing of sequence data were performed using the Quantitative Insights into Microbial Ecology (QIIME2) pipeline (version 2024.5) (26). The sequences were clustered using the DADA2 algorithm (27). The representative sequences from each ASV were aligned to the Greengenes2 reference database (v22.10) (28), and taxonomic classification was performed using the Naive Bayes Classifier. The unclassified ASVs have been filtered out from further downstream analysis. For each data set, the parameters used in the denoising process and the primers used for training the naive Bayes classifier are mentioned in File S2 at https://doi.org/10.5281/zenodo.20175118. The results of the denoising process are described in detail in the Denoising_data sheet of File S3 at https://doi.org/10.5281/zenodo.20175118. The amplicon sequence variants (ASVs) assigned to each genus were summed to generate genus-level abundance information. This genus-level agglomerated data retained an optimal number of ASVs while providing adequate resolution for our analysis (see the Taxonomy_data sheet of File S3 at https://doi.org/10.5281/zenodo.20175118).

Following this, batch correction was performed using meta-analysis methods with a uniform pipeline for heterogeneity in microbiome studies (MMUPHin) (29) after applying a 1% prevalence cutoff and a minimum read count threshold of 50 to reduce project-specific bias in the data set.

### Statistical analysis

All statistical analyses were performed after agglomerating the ASV table at the genus level. The Chao1 and Shannon diversity indices were calculated using the “vegan” package v2.6.10 (30) in R Statistical Computing Environment 4.3.1 (31). The pairwise-Wilcoxon test was performed using the “Microbiome” v.1.24.0 package (32) to test the significance of the differences in Shannon diversity metrics among samples. The input to the test consisted of genus-level alpha diversity values calculated for each sample, grouped according to latitude zones.

All further analyses have been performed after setting a read cutoff of 50, and a prevalence cutoff of 1% as rare taxa can inflate between-sample distances and introduce technical variability without contributing meaningful biological signals. Permutational multivariate analysis of variance (PERMANOVA) was conducted using the “adonis2” function in the “vegan” package (30) to evaluate the contribution of metadata variables to between-sample variation. The analysis was performed on a Bray–Curtis dissimilarity matrix calculated from genus-level relative abundance data, with metadata variables, including Project, sample origin, and latitude zones and ocean from which the sample was collected. Statistical significance was assessed using 999 permutations. The Bray-Curtis dissimilarity index was used as the basis for the principal coordinate analysis (PCoA) and was then used to plot the beta diversity of the samples. Beta dispersion, which shows the spread or variability in the community composition, was estimated using the “betadisper” function. The canonical correspondence analysis (CCA) and variation partition analysis (VPA) were performed using the “vegan” package (30) to corroborate the PERMANOVA results.

Phylum-level abundances were calculated after transforming the data to relative abundance. Differences in relative abundance of phyla across latitude zones were assessed using the Kruskal–Wallis test. The input to the test consisted of relative abundance values (proportion-normalized counts) for each phylum across samples, with samples grouped according to latitude zones (tropical, temperate, and polar).

### Community assembly

The input for all community analyses was the genus-level abundance table generated following denoising of sequencing data using QIIME2, agglomerated at the genus level, and filtered to retain taxa with more than 1% prevalence and a minimum of 50 reads.

The neutral community model (NCM) was used to estimate the contribution of stochastic and deterministic processes in community assembly of marine samples (10). For each genus, the mean relative abundance across samples and its occurrence frequency (proportion of samples in which the genus was detected) were calculated. The model was fitted to describe the relationship between taxon abundance and occurrence frequency, and the migration rate (*m*) was estimated. The product *Nm*, where *N* is the metacommunity size and *m* is the migration rate, was used to represent the effective dispersal rate, reflecting the balance between dispersal and ecological drift. The *R*^2^ value was used to evaluate the extent to which community assembly could be explained by neutral processes, with deviations from model predictions indicating potential deterministic influences. The “MicEco” (33) and “vegan” packages (30) were used for the NCM analysis at the genus level.

Another approach used to reveal the patterns of stochasticity and determinism and their influence on microbial communities was to calculate Levin’s niche breadth (B) index (Bcom) (34). Levin’s niche breadth at the community level (Bcom) represents the average niche breadth of all taxa within a community, reflecting the extent to which taxa are distributed across different environments. Higher Bcom values indicate that taxa are more variable in composition (generalists), suggesting greater ecological flexibility. This analysis was conducted using the “niche.width” function in the “spaa” package available in R (35).

Permutational analysis of multivariate dispersion (PERMDISP) was performed to assess differences in within-group variability in community composition. This analysis was conducted using the “betadisper” function in the “vegan” package (30), based on the Bray–Curtis dissimilarity matrix. PERMDISP calculates the distance of each sample to the centroid of its respective group in multivariate space, and differences in dispersion among groups were tested using permutation-based analysis of variance.

NST was calculated using the NST package in R (v.3.1.10) (36). The normalized stochasticity ratio (NST) quantifies the relative contribution of stochastic and deterministic processes in community assembly. NST values range from 0 to 1, where values >0.5 indicate stochastic processes, while values <0.5 suggest that deterministic processes play a greater role.

A phylogenetic-bin-based framework (iCAMP) was used to infer community assembly mechanisms. Community assembly processes were interpreted based on selection, dispersal, and drift. Homogeneous selection refers to consistent environmental pressures leading to similar community compositions, whereas heterogeneous selection reflects variable conditions resulting in divergent communities. Dispersal limitation reduces taxa exchange between communities, increasing compositional differences, while homogenizing dispersal promotes similarity through high rates of taxa exchange. Drift represents stochastic changes in community composition driven by random demographic processes independent of selection and dispersal. The icamp.big, icamp.bins, and icamp.boot functions in the R package iCAMP were used (11).

### Co-occurrence networks construction and analyses

The microbial co-occurrence network was constructed based on the association values computed using SParse InversE Covariance Estimation for Ecological Association Inference (SPIEC-EASI) (12). A cutoff of at least 50 reads and a minimum of 1% prevalence was used to filter out noise and avoid spurious interactions. Network analysis was performed using the “igraph” (37) and “NetCoMi” packages (38). The plots generated in the study have been generated using the “ggplot” package (39).

Network robustness was assessed by evaluating the response of the network to targeted node removal. Nodes were sequentially removed in order of decreasing degree (highly connected nodes first), and the natural connectivity of the network was recalculated after each removal step. Changes in natural connectivity were used to quantify the resilience of the network, with larger decreases indicating lower robustness to targeted perturbations.

Genera were classified into topological roles based on their within-module connectivity (*Z*-score) and among-module connectivity (participation coefficient [*P*]) following reference 40. The within-module degree *Z*-score (*Z*) quantifies how well a genus is connected to other genera within its module, while the participation coefficient (*P*) measures how evenly its connections are distributed across different modules. Based on threshold values (*Z* = 2.5 and *P* = 0.62), genera were categorized into four roles: peripherals (low *Z*, low *P*), connectors (low *Z*, high *P*), module hubs (high *Z*, low *P*), and network hubs (high *Z*, high *P*).

A Fisher’s exact test was performed to assess whether the distribution of taxa differed significantly between generalists and specialists. The input to the test consisted of contingency tables constructed using the abundance counts of generalist and specialist taxa compared with all other taxa within each phylum. Statistical significance was evaluated using two-sided Fisher’s exact tests.

## RESULTS

### Latitude zones and sample origin contribute significantly to the variation in marine bacterial community

**Fig 1:**
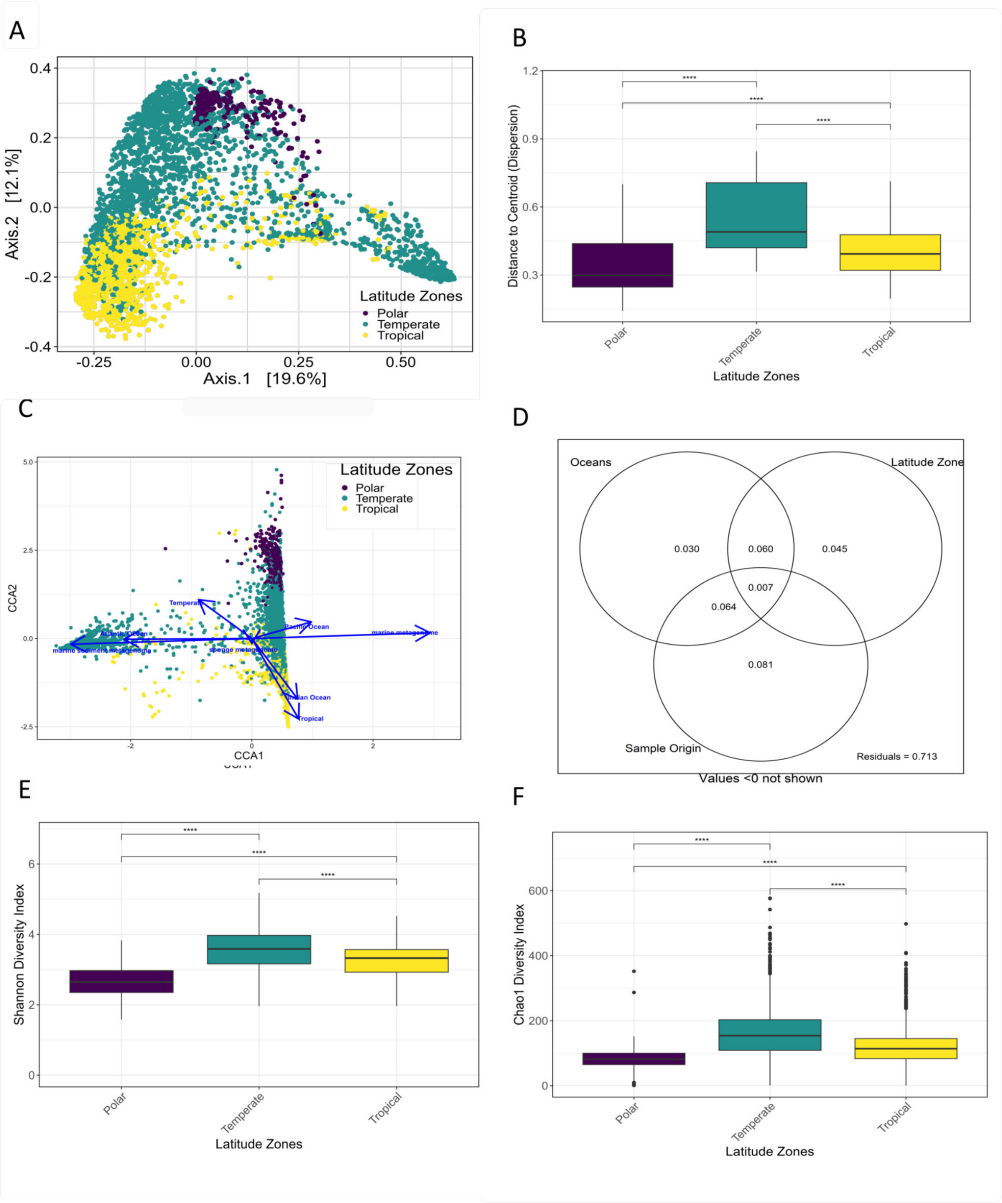
Compositional Analysis of marine microbial communities. (**A**) PCoA. (**B**) Beta-dispersion (pairwise Wilcoxon test, *P*-value < 0.001). (**C**) CCA depicting the effect of different environmental factors on the Bray–Curtis distance. (**D**) VPA. (**E**) Shannon diversity of samples grouped by latitude zones. Polar region 2.64 ± 0.57, temperate region: 3.5 ± 0.74, and tropical region: 3.18 ± 0.7. (F) Chao1 diversity of samples grouped by latitude zones. Polar region 83.33 ± 34.22, temperate region: 161.22 ± 78.13, and tropical region: 119.46 ± 62.81.

To capture the differences in community composition, Bray-Curtis dissimilarity was calculated. PERMANOVA was performed to quantify the extent to which these factors contribute to diversity variation after using MMUPHin to remove project-based bias. PERMANOVA results showed that the latitude zones explain 4.6% and sample origin contributes 6.7% of the variation. The Bray-Curtis distance was ordinated with PCoA. The first two principal axes accounted for more than 30% of the variation (see Fig. S2 at https://doi.org/10.5281/zenodo.20175118). The bacterial communities were clustered into three groups based on the latitude zones (polar, temperate, and tropical; Fig. 1A). The beta dispersion of samples was evaluated to compare microbiome composition variability across different latitude zones (tropical: 0.434 ± 0.174, temperate: 0.545 ± 0.154, and polar: 0.354 ± 0.152). Samples from temperate regions exhibited the highest beta dispersion, followed by those from tropical and polar regions (Fig. 1B). The differences in beta dispersion between latitude zones were statistically significant (pairwise Wilcoxon test *P*-value < 0.001).

CCA revealed that sample origin, latitude zones, and ocean significantly influenced marine bacterial community composition (Fig. 1C). The analysis showed that latitude zones, sample origin and the ocean of origin had significant correlations (*P*-value < 0.05) with the bacterial communities (see Table S3 at https://doi.org/10.5281/zenodo.20175118). Variation partition analysis (VPA) was performed to quantify the relative contributions of latitude, ocean of origin, and sample origin to variation in microbial community composition (Fig. 1D). The ocean of origin explained the largest proportion of variation (16.1%), followed by sample origin (15.2%) and latitude (11.2%). A substantial fraction of the variation (6.7%) was jointly explained by latitude and ocean, reflecting their inherent correlation. The overlap between other variables was comparatively smaller. A large proportion of the variance (71.3%) remained unexplained, suggesting the influence of additional environmental or stochastic factors not captured in the model.

Alpha diversity indices (Shannon and Chao1) were also calculated to complement the beta diversity patterns. ANOVA results and effect size analysis indicated that latitude zones and sample origin are the primary contributors to differences in alpha diversity indices (see Table S4 at https://doi.org/10.5281/zenodo.20175118). The sample origin and the ocean of origin also showed significant differences in all alpha diversity indices *P*-value < 0.001. Polar regions showed the lowest Shannon and Chao1 diversity indices (Fig. 1E and F). The bacterial communities present in the temperate regions had the highest alpha diversity indices (Shannon diversity index: 3.37 ± 0.72 and Chao1 diversity index: 160.01 ± 76.71). Pairwise Wilcoxon tests were performed, which showed significant differences *P*-value < 0.001 in the alpha diversity indices between polar, temperate, and tropical zones (Fig. 1E and F). Based on the contribution of environmental variables to variation in diversity (see Table S2 at https://doi.org/10.5281/zenodo.20175118) we retained latitude and sample origin as the primary environmental variables.

The Kruskal-Wallis test was conducted at the phylum level to assess taxonomic shifts across latitude zones. *Campylobacterota*, *Verrucomicrobiota*, and *Marinisomatota* showed varying abundances across latitude zones (see Table S5 at https://doi.org/10.5281/zenodo.20175118). *Verrucomicrobiota* was relatively more abundant in temperate regions, whereas *Campylobacterota* and *Marinisomatota* were abundant in polar and tropical regions, respectively. *Pelagibacter* was the most abundant genus in the ocean bacterial community *P*-value < 0.001.

PERMANOVA and VPA analyses indicate that the observed variation is primarily explained by sample origin and latitudinal zones; these factors are examined in detail in the following sections.

### Marine communities display high stochastic community assembly processes

**Fig 2:**
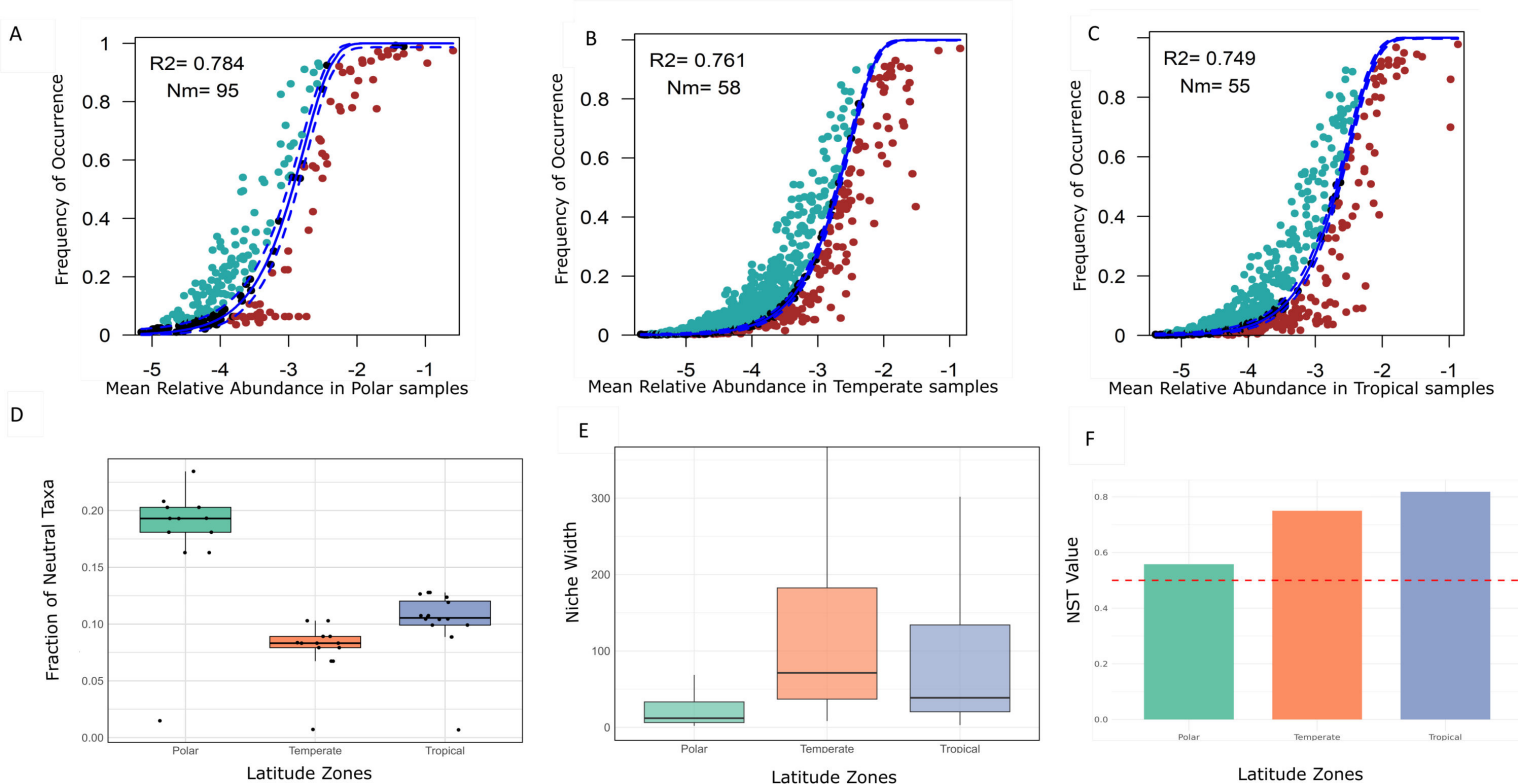
Assessment of neutral model fit across marine microbial communities in polar, temperate, and tropical zones. (**A–C**) Comparison of the fit of polar (**A**), temperate (**B**), and tropical (**C**) marine microbial communities to the neutral community model. The blue line represents the predicted distribution under the neutral model. Taxa colored blue fit the model predictions, while green and red indicate taxa with significantly higher or lower abundances than expected, respectively. (**D**) Fraction of neutral taxa identified in polar, temperate, and tropical marine regions. (**E**) Bcom values illustrating the niche width of taxa across polar, temperate, and tropical communities. (**F**) NST values of the polar, tropical, and temperate regions.

This section investigates the processes driving community assembly in marine water samples across different latitude zones. The NCM analysis was employed to assess the role of neutral processes, such as random dispersal and ecological drift, in shaping the assembly of bacterial communities (Fig. 2A through C). The microbial communities in the tropical regions had the lowest *R*^2^ value ( *R*^2^= 0.749, Nm = 55), and the polar regions had the highest *R*^2^ value ( *R*^2^= 0.784, Nm = 95), indicating the best fit with the neutral model. The estimated dispersal parameter (*Nm*) reflects the combined influence of migration and community size on taxon distribution and was highest in the polar bacterial communities.

Following the NCM analysis, the fraction of taxa that conformed to neutrality was quantified to assess if stochastic processes could explain the community composition. There was a significant difference in the fraction of taxa conforming to NCM across latitude zones (Fig. 2D) (Wilcoxon test: *P*-value < 0.001). The polar (mean: 0.178) and temperate (mean: 0.087) regions exhibited the highest and lowest fraction of neutral taxa, respectively.

A null model analysis was conducted on PERMDISP to examine the variation in community composition across latitude zones. The null model test results showed that dissimilarities in the polar and tropical regions were significantly lower than null expectations (see Table S7 at https://doi.org/10.5281/zenodo.20175118). The temperate regions had a higher dissimilarity than the null expectations, while polar microbial communities have significantly lower dissimilarity, suggesting a more homogenous community composition in polar samples.

We also compared the niche width across latitude zones to assess the factors driving community turnover. Polar communities displayed less variability in Bcom values (Fig. 2E) (standard deviation: 38.8, mean: 29.1), indicating that the community composition is relatively similar across the samples. In contrast, temperate (standard deviation: 137.0, mean: 130.0) and tropical regions displayed significantly more variability in Bcom (standard deviation: 121.0, mean: 97.3), suggesting greater beta-diversity and spatial turnover. NST was calculated to quantify the relative contribution of the stochastic and deterministic processes (Fig. 2F). These results show that stochastic processes dominate across all latitude zones, with the highest NST value observed in tropical regions (mean: 0.81) and the lowest in polar regions (mean: 0.55).

**Fig 3:**
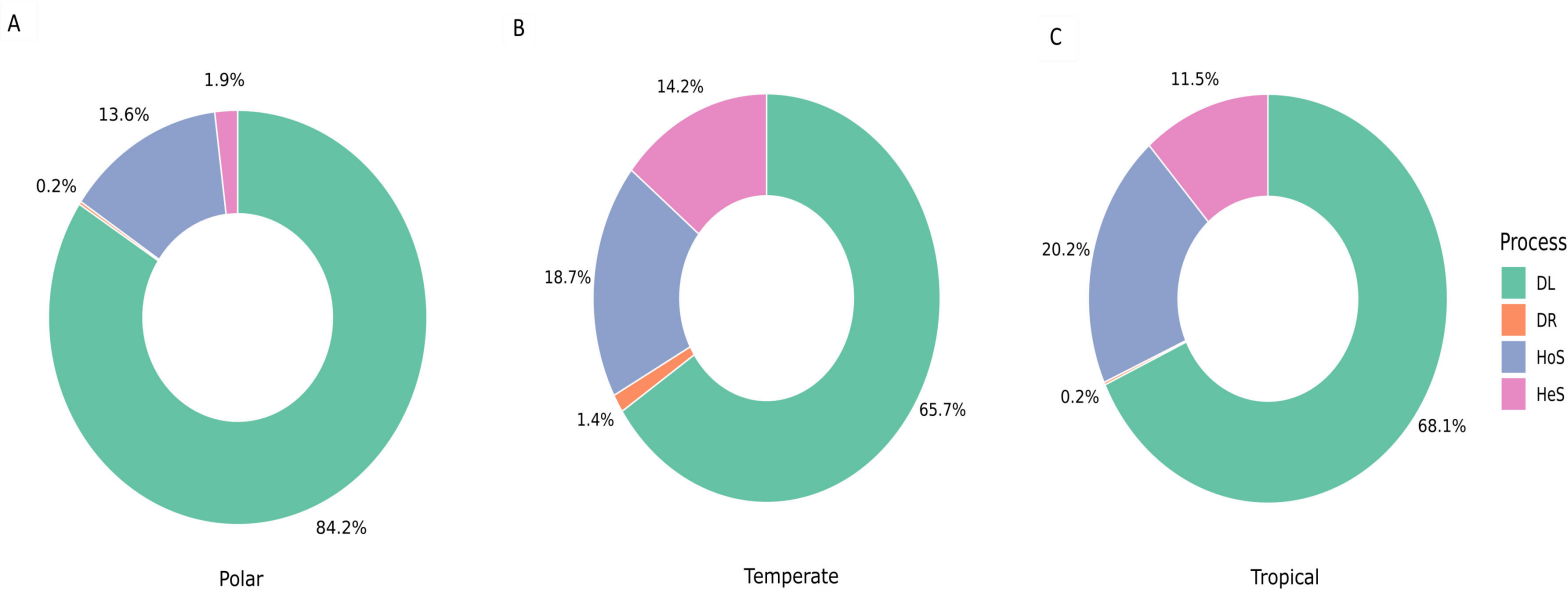
Patterns of community assembly processes across latitudinal marine microbial communities. (**A–C**) Relative contributions of community assembly processes—including dispersal limitation (DL), drift (DR), homogeneous selection (HoS), and heterogeneous selection (HeS)—in (**A**) polar, (**B**) temperate, and (**C**) tropical regions.

We further applied the iCAMP framework to partition stochastic and deterministic processes into specific ecological mechanisms, such as selection, dispersal, and drift, to understand the ecological processes driving community assembly (Fig. 3A through C). Dispersal limitation was the dominant driver of community assembly across all latitude zones, with its influence being particularly pronounced in the polar regions (84.2%). Homogeneous and heterogeneous selection played a relatively more important role in the tropical (HoS 18.7%, HeS: 14.2%) and temperate communities (HoS 20.2%, HeS: 11.5%) than in the polar communities.

These results reveal that bacterial community assembly in the ocean is shaped by both stochastic and deterministic processes, with strong environmental selection playing dominant but latitude-dependent roles.

### Network modularity enhances perturbation resistance in polar microbiomes

We then proceeded to study the co-occurrence patterns using network analysis to understand the ecological interactions shaping these microbial communities in marine water samples. The network constructed using the above methods had 421 nodes and 2,131 edges. Overall, positive interactions predominate (88.6%) in the co-occurrence networks. After removing the negative edges, the modularity of the network was calculated using the Louvain algorithm (41). The network has a modularity of 0.554. Given the modularity value (>0.4), the network has an overall modular structure (42), with eight sub-modules with sizes varying from 19 to 92 nodes. The network has an average degree of 10.12 and an average clustering coefficient of 0.232.

**Fig 4:**
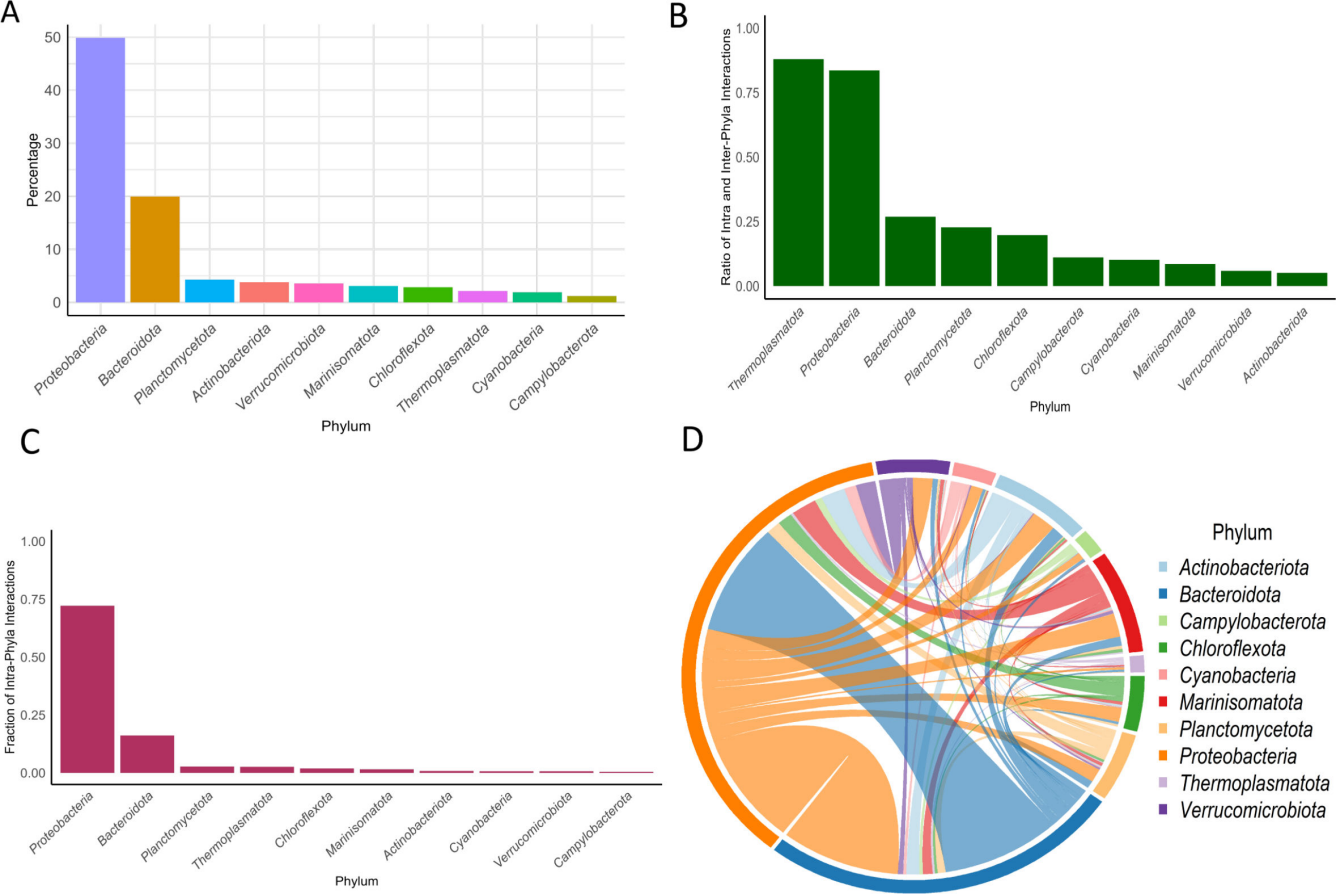
Phylum-level structure and interaction patterns in the ocean microbial community network. (**A**) Distribution of phyla represented by nodes within the ocean microbial community network. (**B**) Ratio of intra-phylum to inter-phylum interactions among nodes, illustrating connectivity between and within different phyla. (**C**) Proportion of intra-phylum interactions attributed to each phylum, highlighting internal network structure. (**D**) Composition and types of inter-phylum interactions across the entire ocean microbial network. The outer ring represent the phyla with different colors representing different phyla, as shown in the legend. The lines represent interactions between the phyla.

Nodes in the co-occurrence network largely belonged to the phyla Proteobacteria (49.8%), Bacteroidota (19.95%), Planctomycetota (4.28%), Actinobacteriota (3.8%), Verrucomicrobiota (3.56%), and Marinisomatota (3.09%) (Fig. 4A). Inter-phylum interactions comprised 1,916 of the total interactions, with the remaining 835 occurring within phyla. Inter-phylum interactions vastly outnumber intra-phylum interactions across all phyla. Thermoplasmota and Proteobacteria exhibited the highest intra-phylum to inter-phylum interaction ratios (Fig. 4B). Proteobacteria and Bacteroidota accounted for 72.2% and 16.1% of all the intra-phylum interactions (Fig. 4C) and had the largest number of inter-phylum interactions (Fig. 4D).

We then constructed latitude-zone-specific co-occurrence networks. The polar, temperate and tropical co-occurrence networks had 185, 429 and 325 nodes. The degree distribution of these networks is shown (see Fig. S3 A through C at https://doi.org/10.5281/zenodo.20175118). The temperate co-occurrence network had the highest average degree of 10.219. The temperate co-occurrence network had the least density (Table 2). The clustering coefficient was highest in the tropical co-occurrence network. The polar, temperate, and tropical co-occurrence networks across latitude zones have a modular structure (42). The modularity is reported to be the highest for the polar co-occurrence network.

**TABLE 2:** Properties of polar, temperate, and tropical microbial co-occurrence networks

Property	Polar	Temperate	Tropical
Number of nodes	185	429	325
Number of edges	427	2,192	1,297
Network density	0.025	0.024	0.025
Average degree	4.616	10.219	7.982
Clustering coefficient	0.225	0.227	0.247
Modularity	0.648	0.525	0.565
Ratio of Negative to Positive Edges	0.1297	0.1722	0.1955

To assess network robustness, we removed high-degree nodes (hubs) and evaluated the resulting changes in natural connectivity, a measure of network resilience. Natural connectivity decreases with increasing percentage of nodes removed (targeted by degree) across all three networks. The slopes of the decline were −0.00055 (polar), −0.00050 (temperate), and −0.00044 (tropical). The relatively less negative slope for the tropical network indicates greater robustness to targeted node removal, whereas the polar network exhibited the steepest decline. Consistently, the polar network showed the lowest natural connectivity and the most pronounced decrease following removal of high-degree nodes (see Fig. S4 at https://doi.org/10.5281/zenodo.20175118).

**Fig 5:**
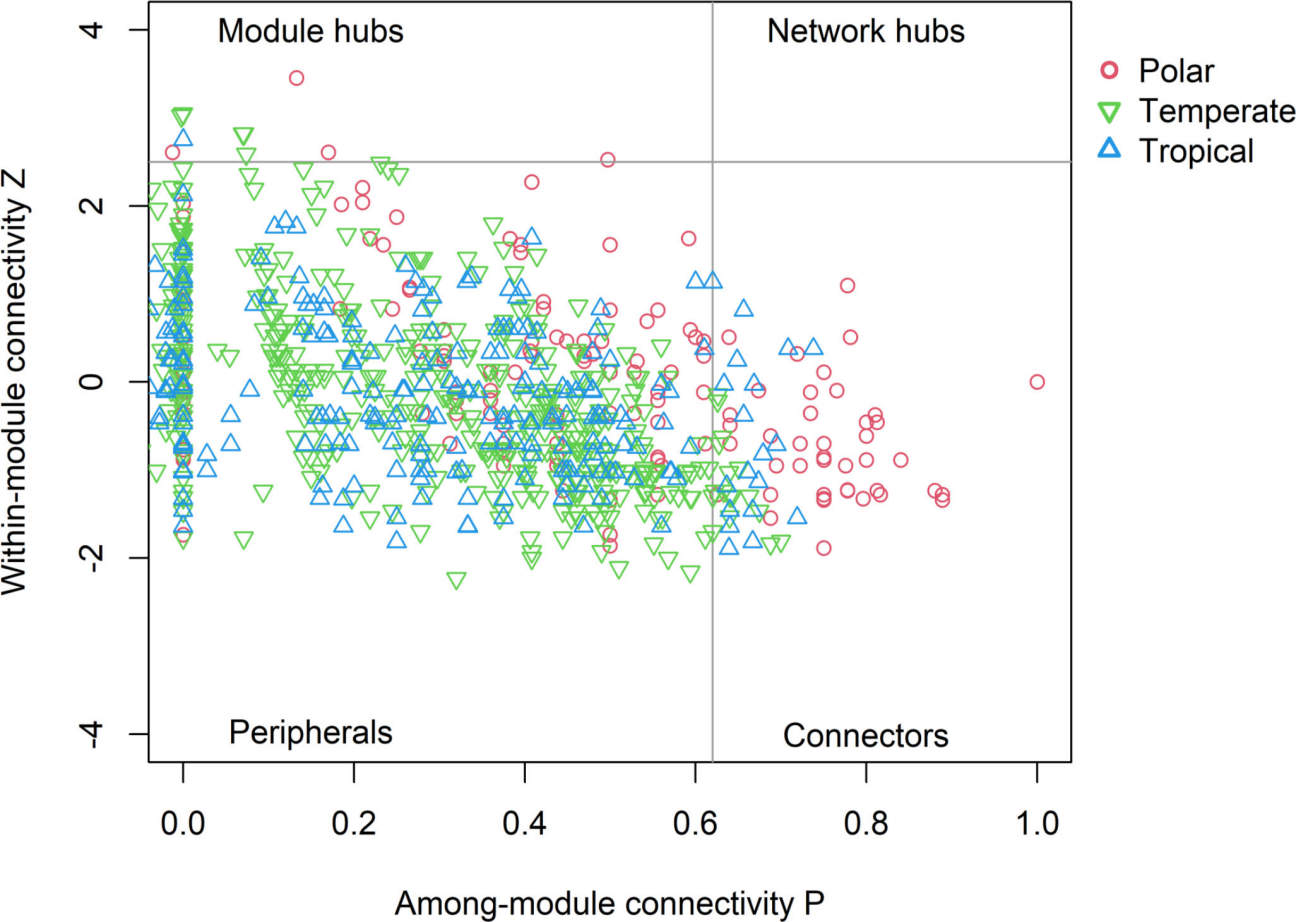
Role of taxa in polar, temperate, and tropical marine microbial communities. Taxa are classified according to their network roles—peripherals, connectors, module hubs, and network hubs—based on patterns of inter- and intra-module connectivity within each co-occurrence network. This highlights the structural and functional positions of taxa across distinct climatic regions.

In addition to the global network properties, the topological properties of the individual genera help understand their ecological roles. Genera are classified into four buckets (Fig. 5) based on their within-module (*Z*) and among-module connectivity (*P*) (40). The polar, temperate, and tropical co-occurrence networks had 2, 7, and 7 module hubs, respectively. The temperate co-occurrence network had the largest percentage of connectors (high *P*, low *Z*) compared with the other co-occurrence networks.

### Specialist taxa play a major role in maintaining the overall structure of the marine microbiome

**Fig 6:**
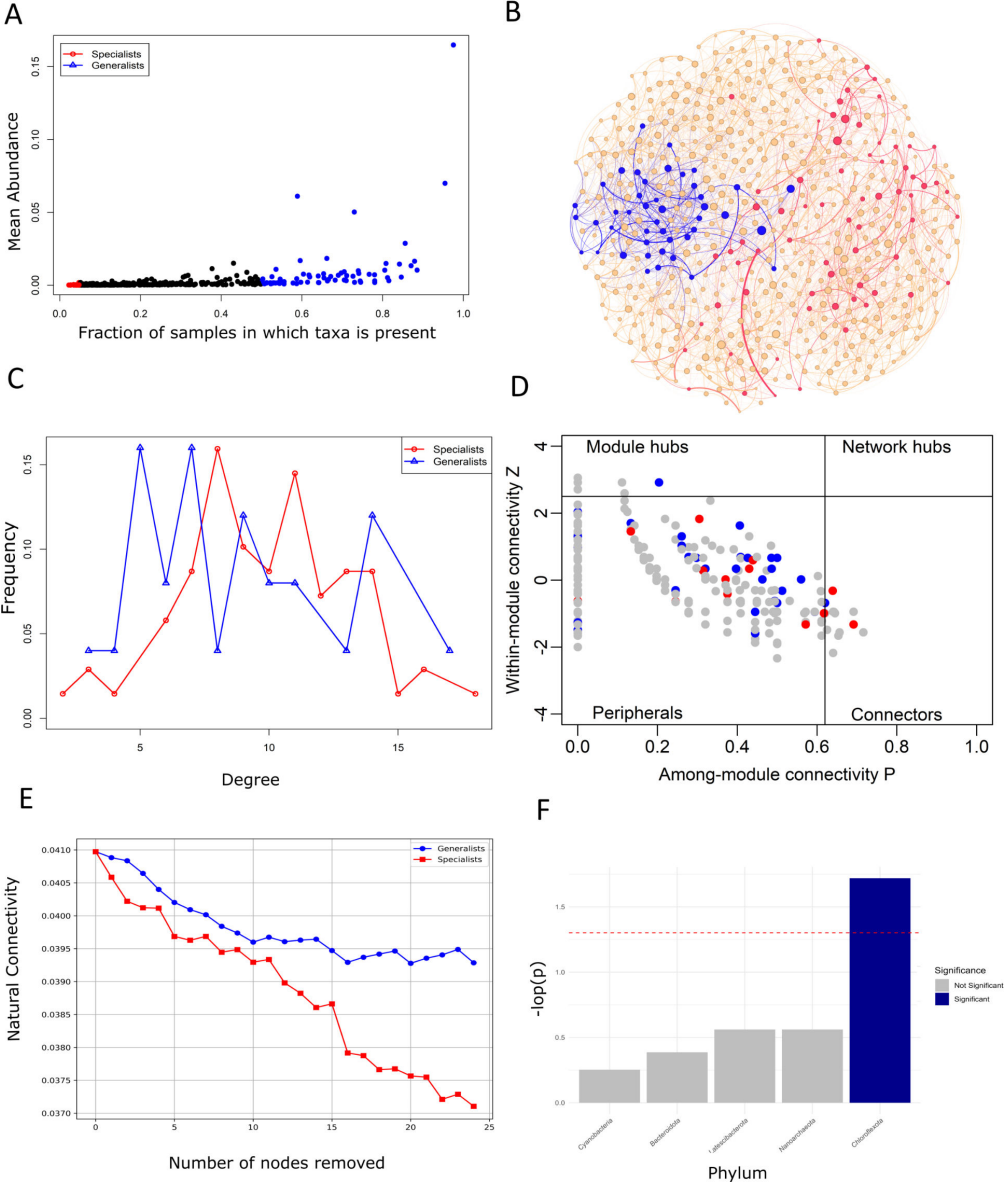
Analysis of the roles of the generalists and specialists in ocean microbial communities. Specialists are shown in red and generalists in blue, while taxa unclassified as generalists or specialists are colored black or gray in panels **A and D**, respectively. (**A**) Occupancy plot depicting the mean relative abundance and prevalence of different taxa in the ocean microbial network. (**B**) Overall ocean microbial co-occurrence network depicting the interactions between generalists and specialists. (**C**) Degree distribution of the generalist and specialist taxa in the ocean microbial network. (**D**) Topological roles of the generalist and specialist taxa in the ocean microbial network based on their inter-module and intra-module connectivity. (**E**) Changes in the natural connectivity of the overall ocean microbial network after removing specialists and generalists to highlight their importance. (**F**) Phyla with a significantly different number of generalists and specialists in the ocean microbial co-occurrence network.

Genera are classified as marine generalists (prevalence > 50%) and specialists (prevalence < 5%) (Fig. 6A). Under this criterion, 15.6% of the genera were classified as generalists, and 5.9% of the genera were classified as specialists. Generalists and specialists tend to interact amongst themselves, rather than with other genera (Fig. 6B). Specialists show a larger variability in the degree compared with the generalists (Fig. 6C). The average degrees of the generalists and specialists were 10 and 8.6, with standard deviations of 3.2 and 3.7, respectively.

To further understand the ecological roles of the generalists and specialists, the inter-module and intra-module connectivity were calculated and plotted (Fig. 6D). Specialists act as connectors. On the other hand, generalists act as module hubs and peripherals. To determine the role of the generalists and specialists on network stability, the natural connectivity of the network was calculated after progressively removing the generalist and specialist nodes of the highest degree. There is a steep drop (generalist slope: −6.6e−05, specialist slope: −1.55e−4) in the natural connectivity of the network upon removal of the specialists compared with removal of the generalists (Fig. 6E).

The generalists belonged to phyla Proteobacteria, Bacteroidota, Actinobacteriota, Cyanobacteria, Marinisomatota, and Verrucomicrobiota. The specialists belonged to a broader range of phyla, including Proteobacteria, Chloroflexota, Bacteroidota, Actinobacteriota, Nitrospirota, and Nanoarchaeota, Nitrospinota, and Thermoproteota. There was a significant difference in the number of generalists and specialists belonging to the phyla Chloroflexota (Fig. 6F) (Fisher test *P*-value < 0.05); 10.41% of the specialists belonged to Chloroflexota, and no generalists were identified in this phylum. Specialists had significantly higher inter-phylum interactions than intra-phylum interactions (Fisher test *P*-value < 0.05). Generalists have nearly equal inter- and intra-phylum interactions (see Fig. S5 at https://doi.org/10.5281/zenodo.20175118).

### Marine sediment-based microbial communities exhibit highest robustness

**Fig 7:**
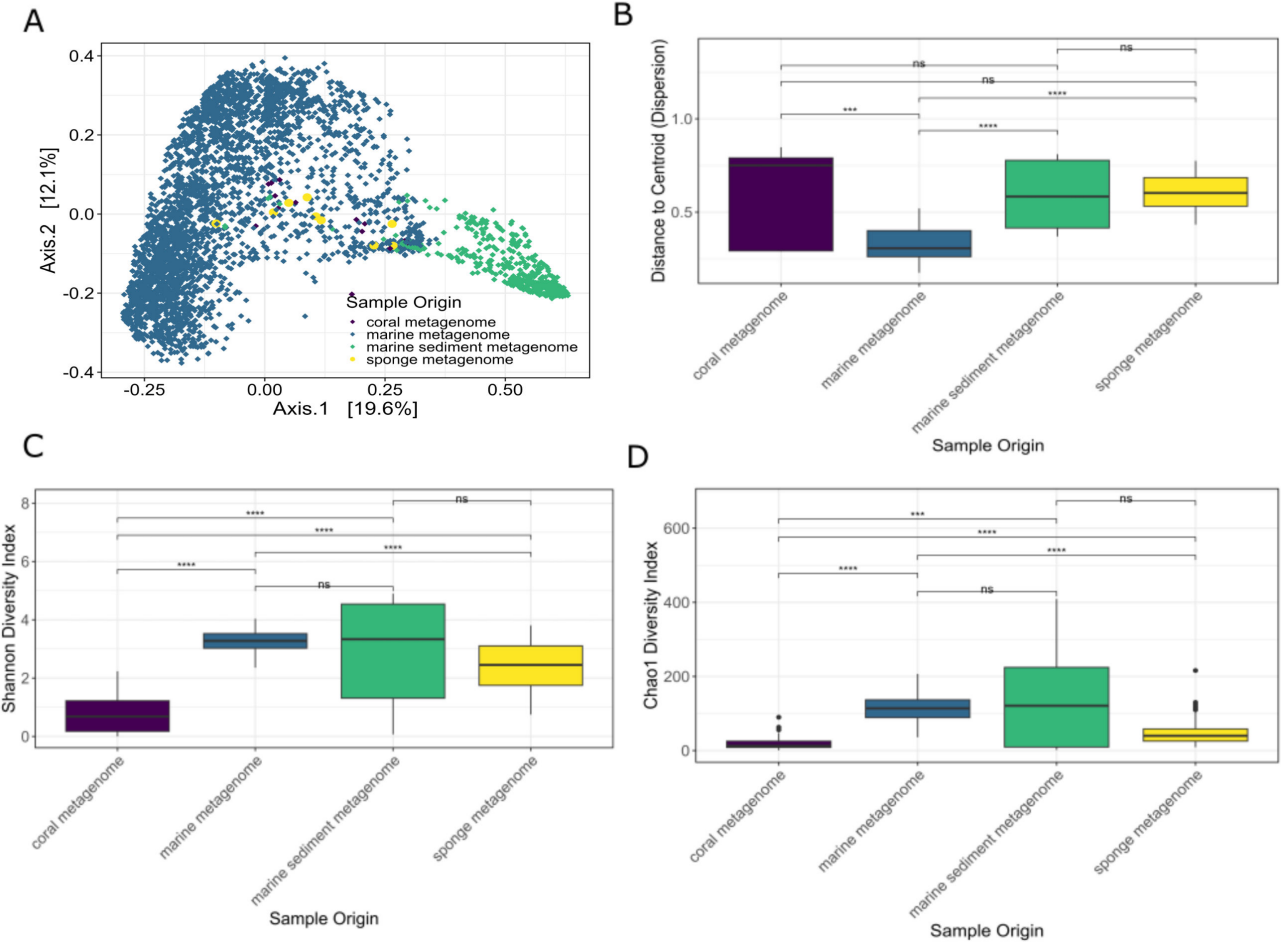
Compositional analysis of marine microbial communities by sample origin. (**A**) PCoA. (**B**) Beta-dispersion (pairwise Wilcoxon test, *P*-value < 0.001). (**C**) Shannon diversity across sample types: coral metagenome (0.81 ± 0.73), marine metagenome (3.23 ± 0.44), marine sediment metagenome (2.93 ± 1.7), and sponge metagenome (2.45 ± 0.75). (**D**) Chao1 diversity across sample types: coral metagenome (19.39 ± 16.86), marine metagenome (113.84 ± 39.97), marine sediment metagenome (136.8 ± 130.66), and sponge metagenome (51.63 ± 40.49).

To further understand and compare the differences in the structure and characteristics of the microbial communities in different marine ecosystems, the Bray-Curtis distance was ordinated with PCoA as shown in Fig. 7A. Since only 51 coral microbial samples were available from tropical regions, we subsampled 51 samples each from marine water, marine sediment, and sponge communities within the same latitudinal zone to ensure equal sample sizes and avoid bias in downstream analyses. The prokaryotic communities were clustered into four groups based on the sample origin (marine metagenome, marine sediment metagenome, coral metagenome, and sponge metagenome). The beta dispersion of samples was evaluated to compare microbiome composition variability across different sample origins (coral: 0.574 ± 0.241, marine metagenome: 0.364 ± 0.172, and marine sediment metagenome: 0.598 ± 0.171, sponge metagenome: 0.604 ± 0.089). Samples from sponge samples exhibited the highest beta dispersion, followed by those from tropical and polar regions (Fig. 7B). The differences in beta dispersion between samples of different sample origin were tested (pairwise Wilcoxon test *P*-value < 0.001).

Alpha diversity indices (Shannon and Chao1) were also calculated to complement the beta diversity patterns. ANOVA results and effect size analysis indicated that sample origin factor showed significant differences in all alpha diversity indices *P*-value < 0.001. Coral samples showed the lowest Shannon and Chao1 diversity indices (Fig. 7C and D). The prokaryotic communities present in the marine samples had the highest Shannon diversity index (Shannon diversity index: 3.23 ± 0.44 and Chao1 diversity index: 160.01 ± 76.71), while marine sediment samples had the highest Chao1 diversity index (Chao1 diversity index: 136.8 ± 130.66, Shannon: 2.93 ± 1.7). Pairwise Wilcoxon tests were performed to identify if the differences between the bacterial communities with different sample origin were significant *P*-value < 0.001 (Fig. 7C and D).

We have not used neutral community models and iCAMP to study the community assembly processes due to limited sample sizes in coral and sponge samples. We studied the co-occurrence patterns using network analysis to understand the ecological interactions shaping these bacterial communities, by constructing co-occurrence networks for samples with different sample origin. The coral-, sponge-, marine-, and marine sediment-based microbial co-occurrence networks have 68, 147, 329 and 477 nodes, respectively (Table 3). The degree distribution of these networks is shown (see Fig. S6A through D at https://doi.org/10.5281/zenodo.20175118). Coral, marine, marine sediment, and sponge microbial communities have a modular structure. However, it is seen that the coral microbial communities have the highest modularity.

**TABLE 3:** Properties of coral, marine, sponge, and sediment microbial co-occurrence networks

Property	Coral	Marine	Sponge	Sediment
Number of nodes	68	329	147	477
Number of edges	61	1,061	282	2,362
Network density	0.027	0.02	0.026	0.021
Average degree	1.794	6.45	3.837	9.904
Clustering coefficient	0.185	0.129	0.178	0.176
Modularity	0.843	0.486	0.679	0.409
Ratio of negative to positive edges	0.034	0.299	0.029	0.239

**Fig 8:**
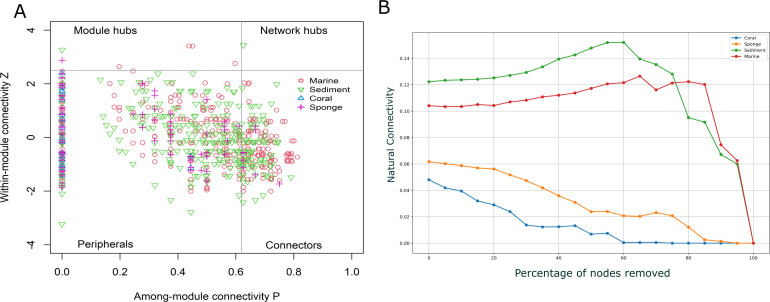
Role of taxa and stability in coral, marine sediment, marine, and sponge microbial communities. (**A**) Taxa are classified according to their network roles—peripherals, connectors, module hubs, and network hubs—based on patterns of inter- and intra-module connectivity within each co-occurrence network. This highlights the structural and functional positions of taxa across distinct climatic regions. (**B**) The variations in the natural connectivity after sequentially removing genera of the highest degree in coral, marine, marine sediment, and sponge networks (color-coded).

In addition to global network properties, node-level topology provides insight into ecological roles (Fig. 8A). Most genera were classified as peripherals across all environments, including sponge (138), coral (64), sediment (391), and marine (260), indicating limited connectivity. Connectors were more prevalent in marine (115), sediment (84), and sponge (8) networks, suggesting stronger inter-module interactions, whereas module hubs were rare (sponge: 1; sediment: 1; marine: 4), and only a single network hub was observed in sediment. Coral networks consisted entirely of peripheral nodes (64). To assess network robustness, we removed high-degree nodes (hubs) and evaluated the resulting changes in natural connectivity. The slopes of the decline were −0.00047 (coral), −0.00068 (sponge), −0.00067 (sediment), and −0.00032 (marine). The relatively less negative slope for the marine network indicates greater robustness to targeted node removal, whereas sponge and sediment networks exhibited the steepest declines, suggesting higher vulnerability. Consistently, sponge and sediment networks showed lower natural connectivity and a more pronounced decrease following removal of high-degree nodes (Fig. 8B), while the marine network maintained comparatively higher stability. Coral networks displayed intermediate robustness.

## DISCUSSION

We structured our study on the latitude variation as it provided the most homogeneous distribution of samples while capturing climatic and environmental gradients. Previous studies have also identified that latitudinal gradients strongly affect the diversity in marine bacterial communities (43–45), with maximum Shannon diversity observed at the temperate regions (Fig. 1E), which is consistent with the intermediate disturbance hypothesis, which proposes that diversity peaks when environmental disturbances occur at moderate levels (46). VPA suggests that the sample origin is a major contributor to bacterial composition. Overall, the three factors, namely latitude, sample origin, and ocean, are highly interrelated variables, and the discrepancies in the batch sizes render meaningful insights challenging.

Diversity analysis at the genus and phylum levels was performed to understand the community composition. *Pelagibacter* is the most dominant genus in the marine samples, consistent with its well-established role as a key component of marine microbial ecosystems (47). While this approach sheds light on key ecological mechanisms, the utilization of 16S rRNA data, results in limited species-level resolution.

The relative contributions of the deterministic and stochastic processes were estimated across different latitude zones. The neutral theory model, null theory model and iCAMP frameworks were used to understand the roles of selection, dispersal and drift in shaping the structure of the marine bacterial communities (11, 34). These three approaches integrate taxonomic community composition, dispersion metrics, and phylogenetic analyses, respectively, to provide a holistic understanding of marine microbial community assembly across latitude zones.

The NCM analysis showed that the dispersal parameter (Nm) was highest in polar communities (Fig. 2A), which also exhibited the greatest proportion of neutral taxa. Consistently, iCAMP identified dispersal limitation as the dominant assembly process (Fig. 3A) (48). Extreme cold and seasonal freeze–thaw cycles likely reduce diversity (Fig. 1E and F) (49), leading to smaller communities where dispersal limitation is more influential (50). However, PERMDISP and Bcom indicate increased community homogeneity, suggesting strong environmental selection.

In temperate regions, stochasticity played a greater role, reflected in higher NCM *R*^2^ and NST values. Elevated Bcom and increased dissimilarity from null expectations indicate high spatial turnover. iCAMP (Fig. 3B) further showed contributions from both homogeneous and heterogeneous selection at the bin level (51). Overall, environmental selection and niche differentiation appear to be key drivers of community assembly in temperate systems.

Tropical communities exhibit a balance of stochastic and deterministic processes driven by high environmental heterogeneity. Elevated Bcom (Fig. 2E) and PERMDISP indicate broad niche differentiation and spatial turnover, while iCAMP (Fig. 3C) highlights contributions from both homogeneous and heterogeneous selection. Together, these results suggest that environmental complexity promotes niche structuring, with stochasticity shaping relative abundances within niches.

Co-occurrence networks were constructed to investigate the ecological interactions and structural organization underlying the observed patterns. The polar regions displayed the highest modularity of 0.648 across latitude zones (Table 2). Higher modularity is a characteristic of a more stable community, as perturbations affecting taxa in one module are less likely to affect the rest of the network (52). The taxa acting as connectors and module hubs are specifically important for maintaining the network structure and stability. Polar bacterial communities displayed the least fraction of connectors and module hubs (Fig. 5). The natural connectivity analysis (see Fig. S4 at https://doi.org/10.5281/zenodo.20175118) revealed that the polar communities are highly vulnerable to hub removal, as they exhibit the most negative slopes. While high modularity in the polar communities provides local stability, the high dependence on fewer connector genera creates systemic vulnerabilities (53). The taxa acting as connectors in the polar microbial co-occurrence network include *Moritella*, which require low temperatures for survival and can degrade complex organic material. The increased vulnerability of polar communities due to greater modularity, along with the nature of connector taxa, suggests they may face risks from warming temperatures (54).

The results suggest that polar microbial communities are shaped by strong spatial structuring and restricted connectivity rather than broad community mixing. Although higher Nm and a greater fraction of neutral taxa may suggest a stronger neutral component, lower NST, higher dispersal limitation in iCAMP, and reduced network connectivity indicate organization into relatively isolated modules (Table 2). In contrast, tropical and temperate communities are shaped by a combination of stochasticity and stronger environmental selection rather than spatial compartmentalization. This is supported by lower Nm, higher NST, reduced dispersal limitation, and stronger homogeneous and heterogeneous selection in iCAMP, along with greater beta dispersion and a more connected, less modular network structure (Table 2).

While the latitude-specific networks provided valuable insights into how community structure and stability vary across latitude gradients, it is also vital to understand the global ocean microbial co-occurrence network to investigate the interaction patterns and phylum-level organization. This allowed for the improvement of the understanding of the ecological trends, such as dominant phyla, the nature of the inter- and intra-phylum interactions. Over 60% of the genera in the global ocean network belong to Proteobacteria and Bacteroidota, (Fig. 4A) consistent with findings from earlier ocean microbiome studies (17, 55–60). The ocean microbiome had higher inter-phylum interactions compared with intra-phylum interactions (Fig. 4B) across nodes. This aligns with studies that suggest that functional complementarity, rather than phylogenetic relatedness, governs ecological interactions in bacterial communities (61, 62).

Specialists act as module hubs and connectors (Fig. 6D), underscoring their role as keystone taxa in maintaining network structure and stability. Despite their widespread prevalence, the generalists act as peripherals, indicating that they interact broadly but weakly, or exist transiently in the community, as evidenced by the stochastic community assembly. To further understand the role of the specialists in network stability, the natural connectivity of the network was computed after removing a fraction of the specialists and generalists (Fig. 6E). The specialists contribute more to the network stability than the generalists. The asymmetric vulnerability suggests that conservation of the specialist taxa is critical for sustaining marine microbial communities under perturbations, such as warming and nutrient shifts, and shows that even rare taxa warrant conservation attention.

Marine sediment communities exhibited the highest Chao1 diversity (136.8 ± 130.66), followed by marine water (113.84 ± 39.97), indicating larger and more heterogeneous species pools (Fig. 7D). Marine communities showed the highest Shannon diversity (3.23 ± 0.44), suggesting more even taxon distributions, whereas coral communities had the lowest Shannon (0.81 ± 0.73) and Chao1 (19.39 ± 16.86) values (Fig. 7C and D), indicative of reduced richness and dominance by few taxa. Although overall trends in diversity and beta-dispersion were observed across sample origins (Fig. 7B through D), not all pairwise differences were statistically significant, suggesting partial overlap in community structure and variability across environments. This indicates that while sample origin influences diversity, stochastic variation and shared taxa may dampen strong differentiation. Coral microbial networks exhibited the highest modularity (0.843; Table 3), suggesting strong compartmentalization that can buffer localized disturbances. However, Zi–Pi analysis (Fig. 8A) revealed that coral networks consist entirely of peripheral nodes, lacking connectors and hubs, unlike marine, sediment, and sponge networks. The presence of connectors and hubs in these systems enhances robustness by maintaining inter-module connectivity and providing alternative interaction pathways, which is consistent with the higher robustness observed in marine networks (Fig. 8B), while sponge and sediment networks remain more vulnerable. Together, these results suggest that modularity alone does not guarantee robustness and that the distribution of topological roles plays a critical role in determining network robustness. Future research could utilize temporal data to enhance understanding of marine microbiome resilience and functional dynamics under ongoing climate change.

## Data Availability

The accession codes for the data sets used in the study are PRJEB42019, PRJEB36282, PRJEB36283, PRJEB25224, PRJNA656268, PRJNA1122929, PRJEB40762, PRJEB45011, and PRJEB27154. All codes supporting the analyses in this study are available in the GitHub repository: https://github.com/RamanLab/community_assembly_for_marine_microbiome. All supplemental information supporting the analyses presented in this study is available at the following Zenodo link: https://doi.org/10.5281/zenodo.20175118.
